# Correction: CDKL3 promotes osteosarcoma progression by activating Akt/PKB

**DOI:** 10.26508/lsa.202201656

**Published:** 2022-10-21

**Authors:** Aina He, Lanjing Ma, Yujing Huang, Haijiao Zhang, Wei Duan, Zexu Li, Teng Fei, Junqing Yuan, Hao Wu, Liguo Liu, Yueqing Bai, Wentao Dai, Yonggang Wang, Hongtao Li, Yong Sun, Yaling Wang, Chunyan Wang, Ting Yuan, Qingcheng Yang, Songhai Tian, Min Dong, Ren Sheng, Dongxi Xiang

**Affiliations:** 1 Department of Oncology, Shanghai Jiaotong University Affiliated Sixth People’s Hospital, Shanghai, PR China; 2 Department of Urology, Boston Children’s Hospital, Harvard Medical School, Boston, MA, USA; 3 College of Life and Health Sciences, Northeastern University, Shenyang, PR China; 4 School of Medicine and Centre for Molecular and Medical Research, Deakin University, Waurn Ponds, Australia; 5 Department of Pathology, Shanghai Jiaotong University Affiliated Sixth People’s Hospital, Shanghai, PR China; 6 Department of Vascular Biology, Boston Children’s Hospital, Boston, MA, USA; 7 Department of General Surgery, Xinhua Hospital Affiliated to Shanghai Jiao Tong University School of Medicine, Shanghai, China; 8 Shanghai Center for Bioinformation Technology and Shanghai Engineering Research Center of Pharmaceutical Translation, Shanghai Industrial Technology Institute, Shanghai, PR China; 9 Department of Orthopedics, Shanghai Jiaotong University Affiliated Sixth People’s Hospital, Shanghai, PR China; 10 Division of Genetics, Department of Medicine, Brigham and Women’s Hospital, Boston, MA, USA; 11 Department of Medicine, Harvard Medical School, Boston, MA, USA; 12 Shanghai Research Center of Biliary Tract Disease Affiliated to Shanghai Jiao Tong University School of Medicine, Shanghai, China

## Abstract

This study demonstrates that CDKL3 regulates Akt activation and its downstream targets to promote OS progression, creating a therapeutically targetable vulnerability in treatment of OS.

Article: He A, Ma L, Huang Y, Zhang H, Duan W, Li Z, Fei T, Yuan J, Wu H, Liu L, Bai Y, Dai W, Wang Y, Li H, Sun Y, Wang Y, Wang C, Yuan T, Yang Q, Tian S, Dong M, Sheng R, Xiang D (2020 Mar 31) CDKL3 promotes osteosarcoma progression by activating Akt/PKB. Life Sci Alliance 3(5): e202000648. doi: 10.26508/lsa.202000648. PMID: 32234750.

In the published article, there were three errors:

1. We designed the primers for PDX1 using GenBank entry AF049893.1. This sequence was entered in 1997 as human Pdx1, but it is actually rat Pdx1. We were unaware of this error and observed a negligible effect on PDX1, so we removed PDX1 from Fig 5B. This does not have any effect on the other conclusions in Fig 5B.

**Figure 5.**
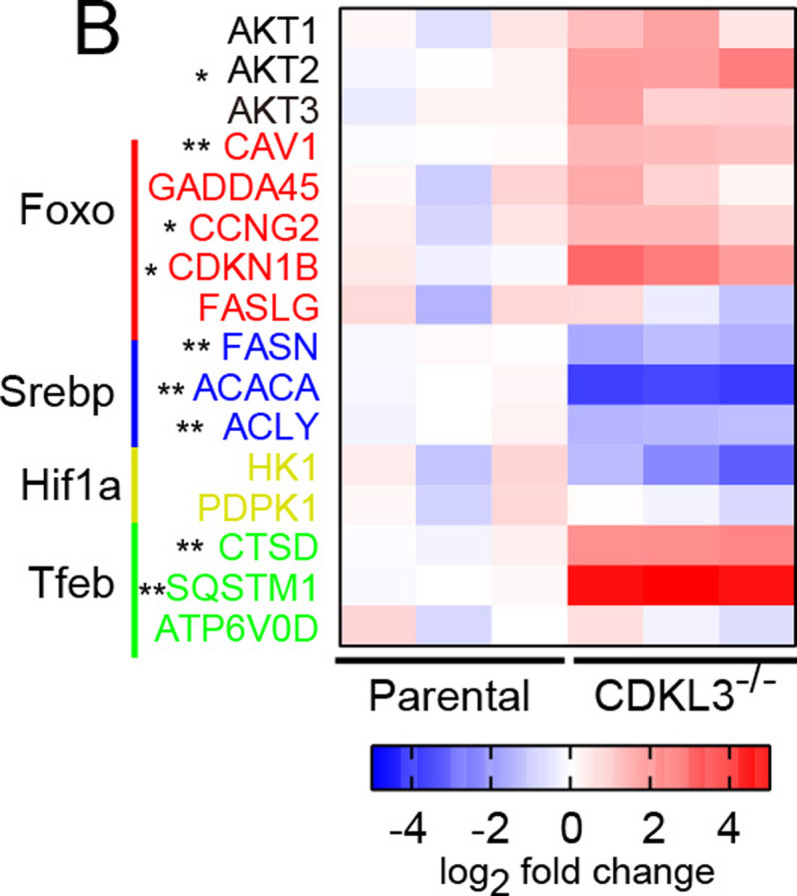
**(B)** The expression patterns of mTORC1 and FoxO downstream target genes confirm that CDKL3 regulates both pathways at the transcription level. Cells were cultured under the regular growth condition.

2. In Fig 6C, lower row, patient (a) the exact CDKL3-staining image was placed back.

**Figure 6.**
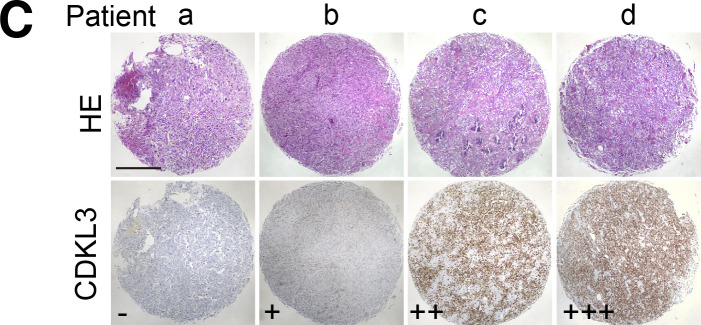
**(C)** Representative immunohistochemistry (IHC) images of OS biopsies with different levels of CDKL3 expression on an OS microarray containing 152 primary OS tissues samples. Scale bar = 500 μm.

3. On page 10, “The insert with triple-HA tag at their N termini (with GSGSGSEF as linker) was further cloned to pcDNA3.1 vector via Gibson Assembly (2621; New England Biolabs) and sequenced using primers 5′-GGGCTGATAAAGAAGGCAGAAAATT;

5′-TGCCTTGGCCTCCCAAAGTGCA.” The correct primers should read as follows: 5′-CGCAAATGGGCGGTAGGCGTG; 5′-GCTGGGGTAGTTCTTCCTCAAGTTC.

The conclusions of this article remain unchanged.

## Supplementary Material

Reviewer comments

