# Supplementary material for: CDKL3 promotes osteosarcoma progression by activating Akt/PKB
Source: Life Sci Alliance. 2020 Mar 31;3(5):e202000648. doi: 10.26508/lsa.202000648 (PMC7119369; doi:10.26508/lsa.202000648)
Supplement: Supplementary file 1 [file LSA-2020-00648_TableS1.docx]

**Supplementary Table 1.** Oligonucleotides information

|  | Targeting sequence (5′→3′) | Sense (5′→3′) | Antisense (5′→3′) |
| --- | --- | --- | --- |
| **Sramble siRNA** |  | GAACGUGACACGUUCGGAGAA | CUCCGAACGUGUCACGUUCUC |
| **CDK6 siRNA#1** | GACAGAGAAACCAAACTAACTTT | AGUUAGUUUGGUUUCUCUGUC | CAGAGAAACCAAACUAACUUU |
| **CDK6 siRNA#2** | ATCAAGACTTGACCACTTACTTG | AGUAAGUGGUCAAGUCUUGAU | CAAGACUUGACCACUUACUUG |
| **CDKL1 siRNA#1** | TTGTTTTCAAATGTAGAAACAGG | UGUUUCUACAUUUGAAAACAA | GUUUUCAAAUGUAGAAACAGG |
| **CDKL1 siRNA#2** | GTCAGATTGTGGCCATCAAGAAG | UCUUGAUGGCCACAAUCUGAC | CAGAUUGUGGCCAUCAAGAAG |
| **CDKL2 siRNA#1** | GAGATATAAAGCCAGAGAATATA | UAUUCUCUGGCUUUAUAUCUC | GAUAUAAAGCCAGAGAAUAUA |
| **CDKL2 siRNA#2** | GTCAAGCTATGCGATTTTGGATT | UCCAAAAUCGCAUAGCUUGAC | CAAGCUAUGCGAUUUUGGAUU |
| **CDKL3 siRNA#1** | AAGTTTTTAGACAGAAAAAGAAA | UCUUUUUCUGUCUAAAAACUU | GUUUUUAGACAGAAAAAGAAA |
| **CDKL3 siRNA#2** | AAGAAAATTCATTTGGTATTTGA | AAAUACCAAAUGAAUUUUCUU | GAAAAUUCAUUUGGUAUUUGA |
| **CDKL4 siRNA#1** | TTGTATTCAAATGCAGAAACAAA | UGUUUCUGCAUUUGAAUACAA | GUAUUCAAAUGCAGAAACAAA |
| **CDKL4 siRNA#2** | AAGAAAATAGCACTAAGAGAAAT | UUCUCUUAGUGCUAUUUUCUU | GAAAAUAGCACUAAGAGAAAU |
| **CDKL5 siRNA#1** | TTCCTAACATTGGTAATGTGATG | UCACAUUACCAAUGUUAGGAA | CCUAACAUUGGUAAUGUGAUG |
| **CDKL5 siRNA#2** | CACAAGGAAACACATGAAATTGT | AAUUUCAUGUGUUUCCUUGUG | CAAGGAAACACAUGAAAUUGU |
| **CDKL3 shRNA** | GGAGATATCTCAGAACCAA | ccgggaGGAGATATCTCAGAACCAActcgagTTGGTTCTGAGATATCTCCtctttttg |  |
| **Sramble shRNA** | TTCTCCGAACGTGTCACGT | ccgggaTTCTCCGAACGTGTCACGTctcgagACGTGACACGTTCGGAGAAtctttttg | CONTROL |
| **CDKL3 gRNA** | GGGCTGTATGATCATTGAGA | caccgGGGCTGTATGATCATTGAGA | aaacTCTCAATGATCATACAGCCCc |
| **CDKL3 (mu)** | Quick change | TGGAAAACCTGTGGATATCTGGGCTTTA(G)G  GA(C)TGC(T)ATGATT(C)ATC(T)GAA(G)ATGGCCACTGGAAATCCCTATCTTCC |  |
