# Supplementary material for: CDKL3 promotes osteosarcoma progression by activating Akt/PKB
Source: Life Sci Alliance. 2020 Mar 31;3(5):e202000648. doi: 10.26508/lsa.202000648 (PMC7119369; doi:10.26508/lsa.202000648)
Supplement: Supplementary file 2 [file LSA-2020-00648_SdataFS2B.ppt]

## Slide 1
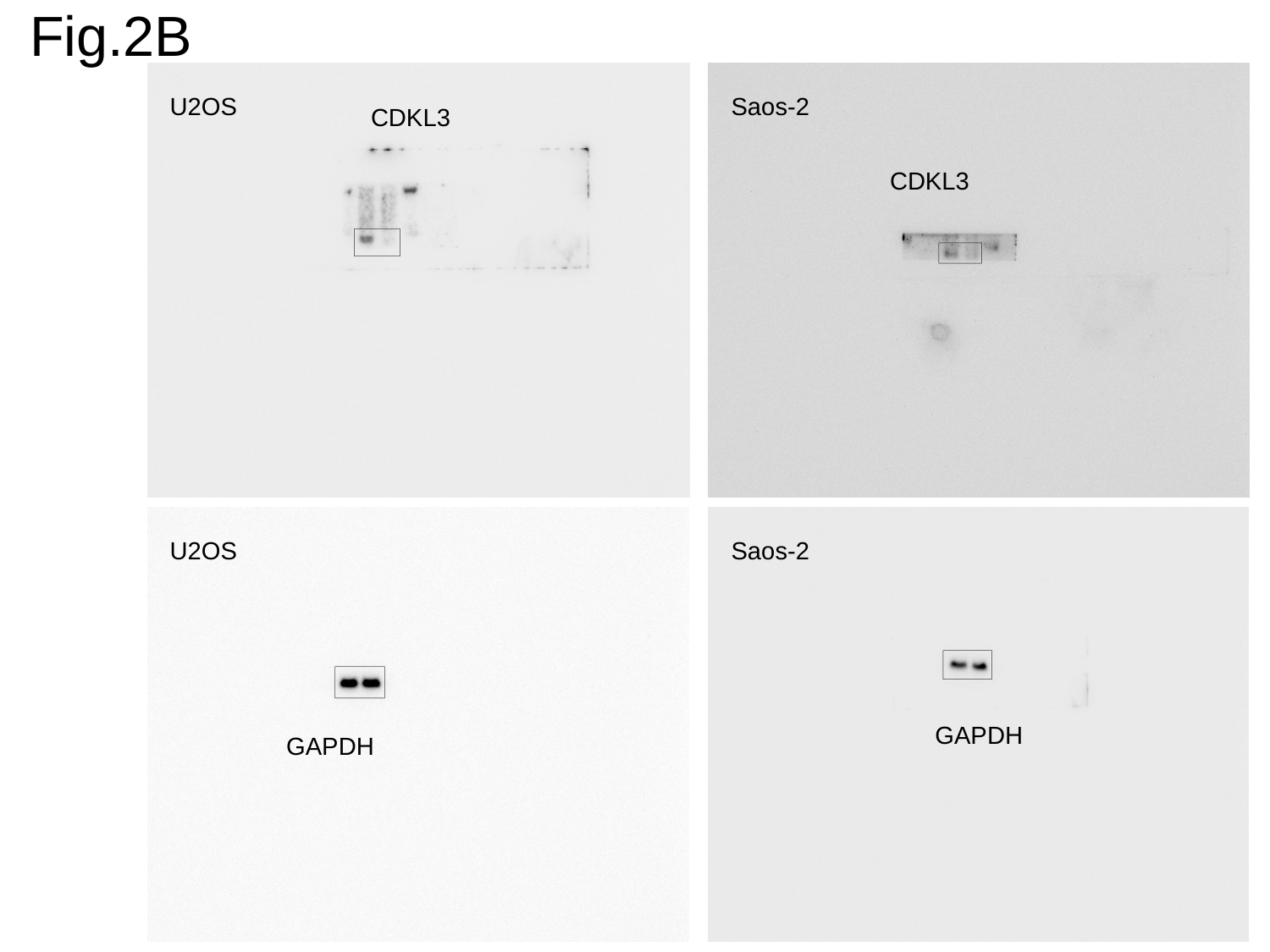

# Fig.2B
U2OS
Saos-2
CDKL3
CDKL3
U2OS
Saos-2
GAPDH
GAPDH

## Slide 2
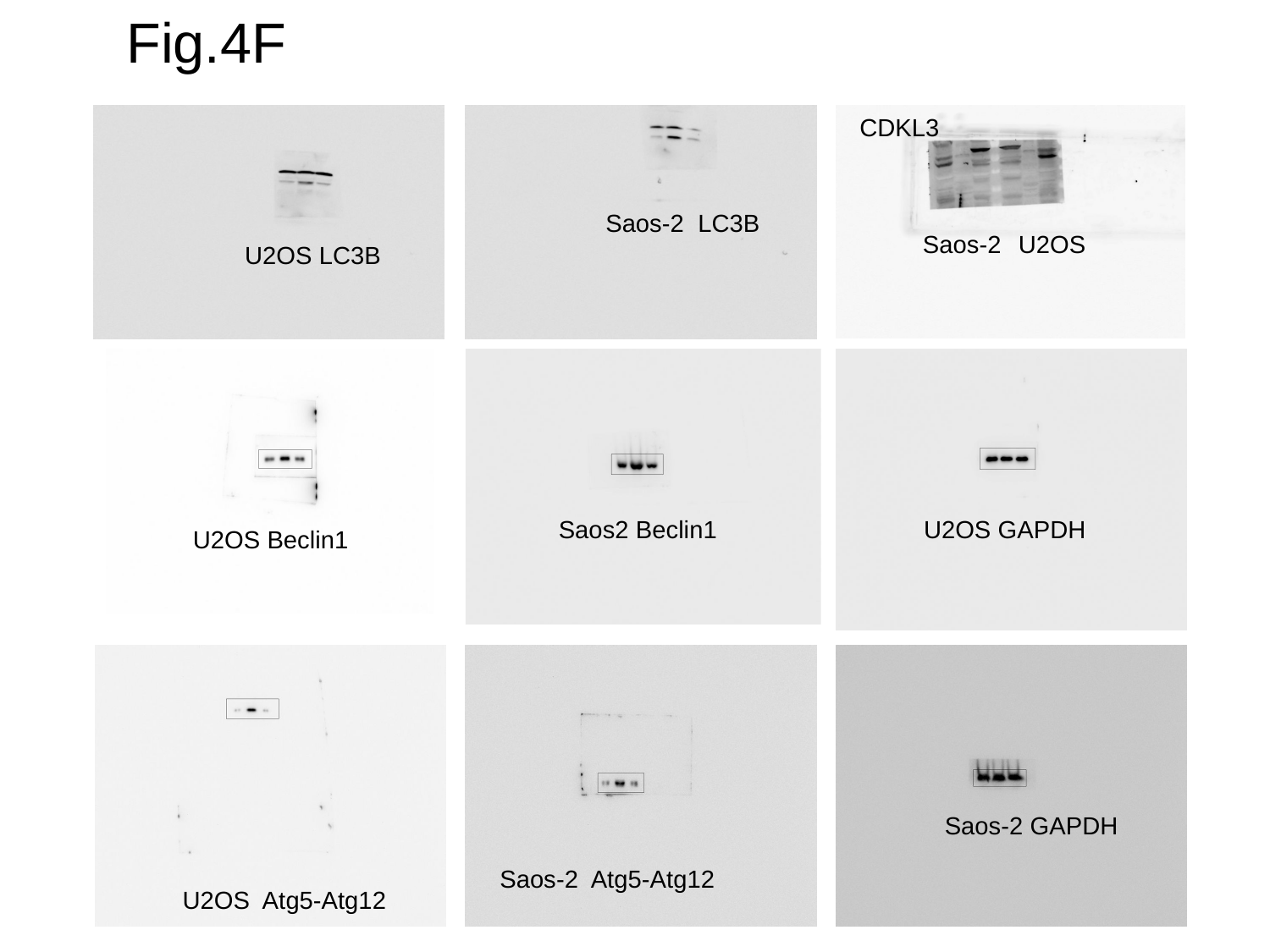

Fig.4F
U2OS LC3B
Saos-2 LC3B
CDKL3
Saos-2
U2OS
Saos2 Beclin1
U2OS GAPDH
U2OS Beclin1
Saos-2 GAPDH
Saos-2 Atg5-Atg12
U2OS Atg5-Atg12

## Slide 3
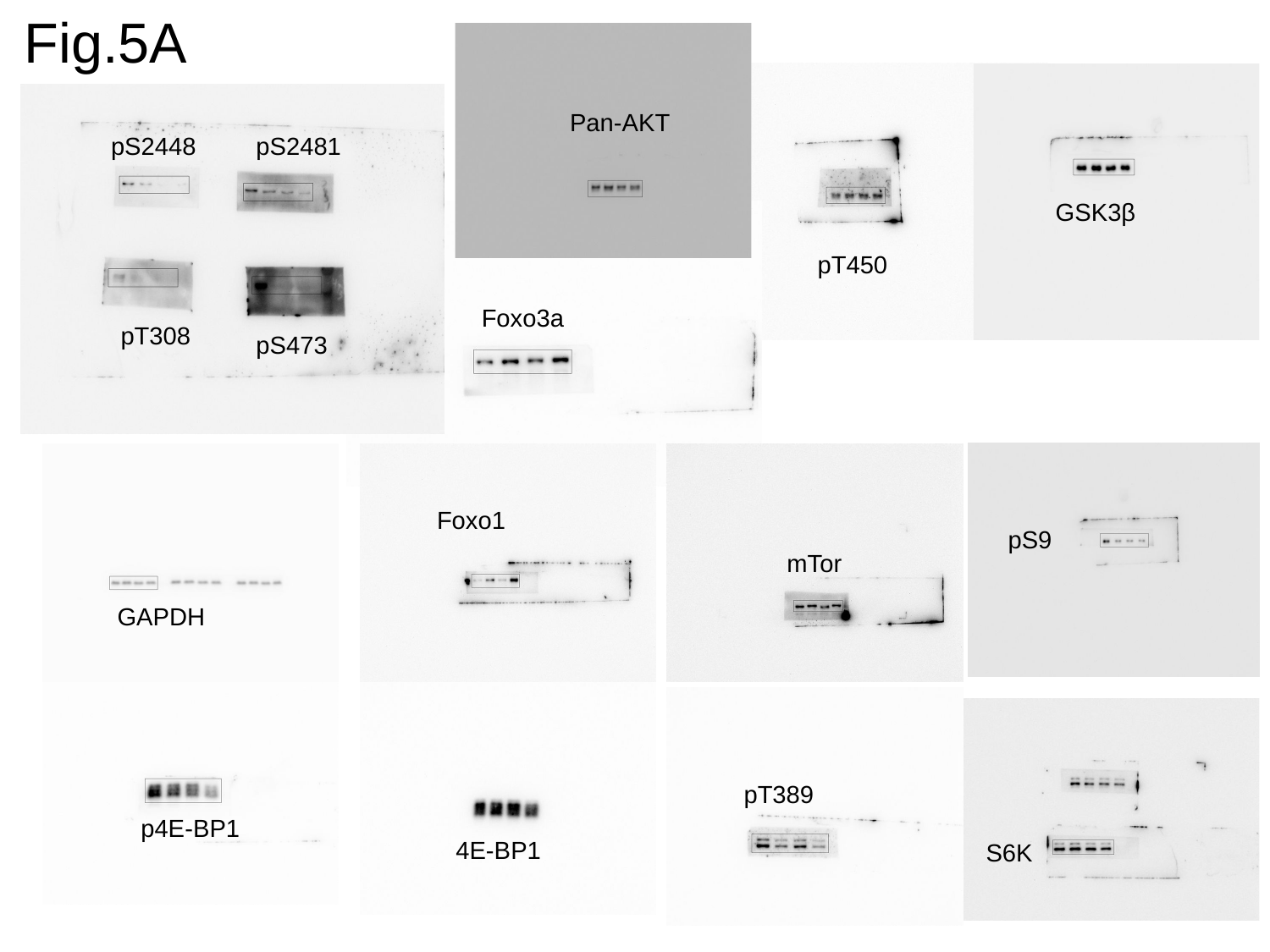

Fig.5A
Pan-AKT
pS2448
pS2481
pT308
pS473
GSK3β
Foxo3a
pT450
GAPDH
Foxo1
mTor
pS9
p4E-BP1
4E-BP1
pT389
S6K

## Slide 4
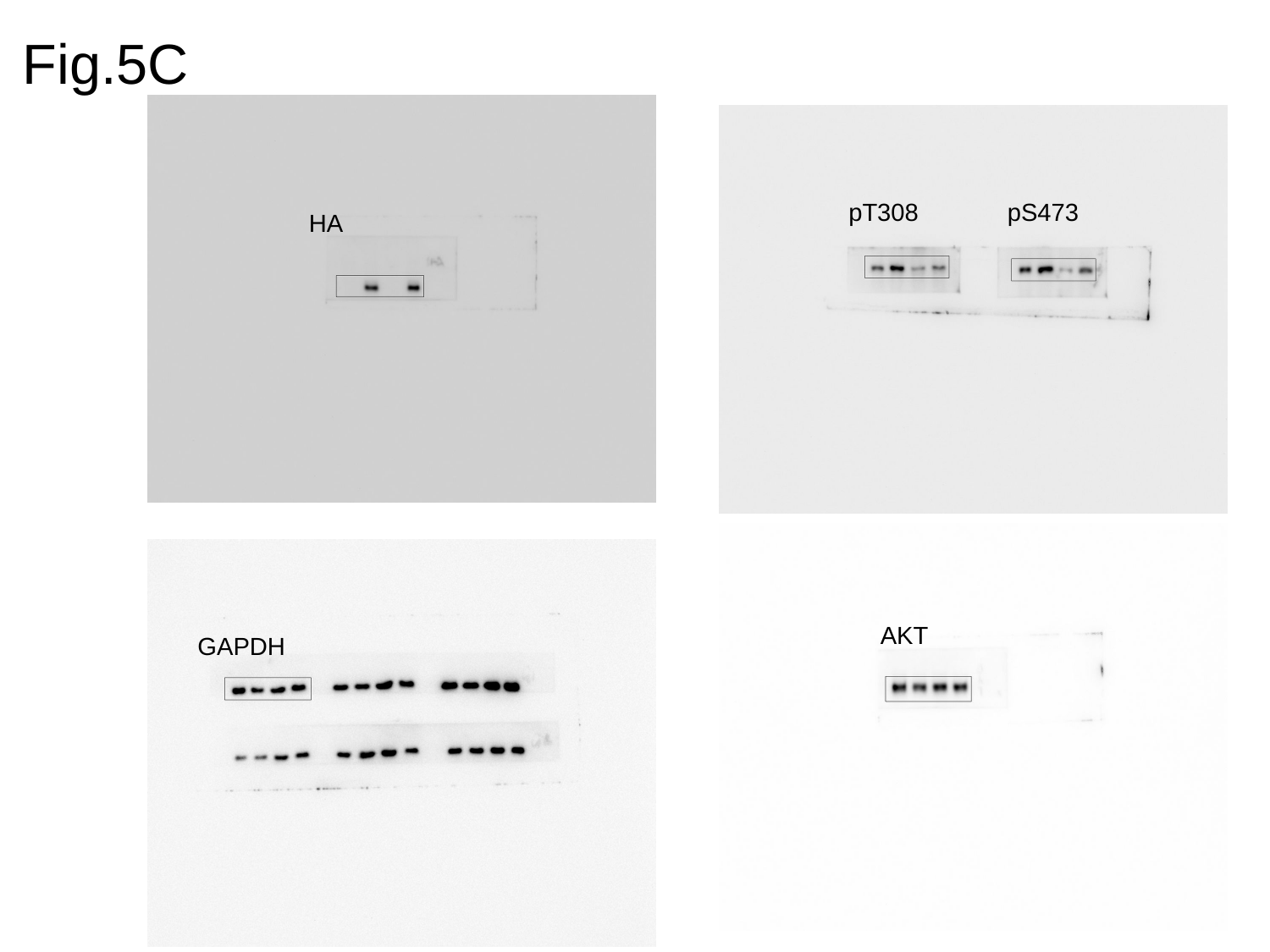

Fig.5C
pT308
pS473
HA
AKT
GAPDH

## Slide 5
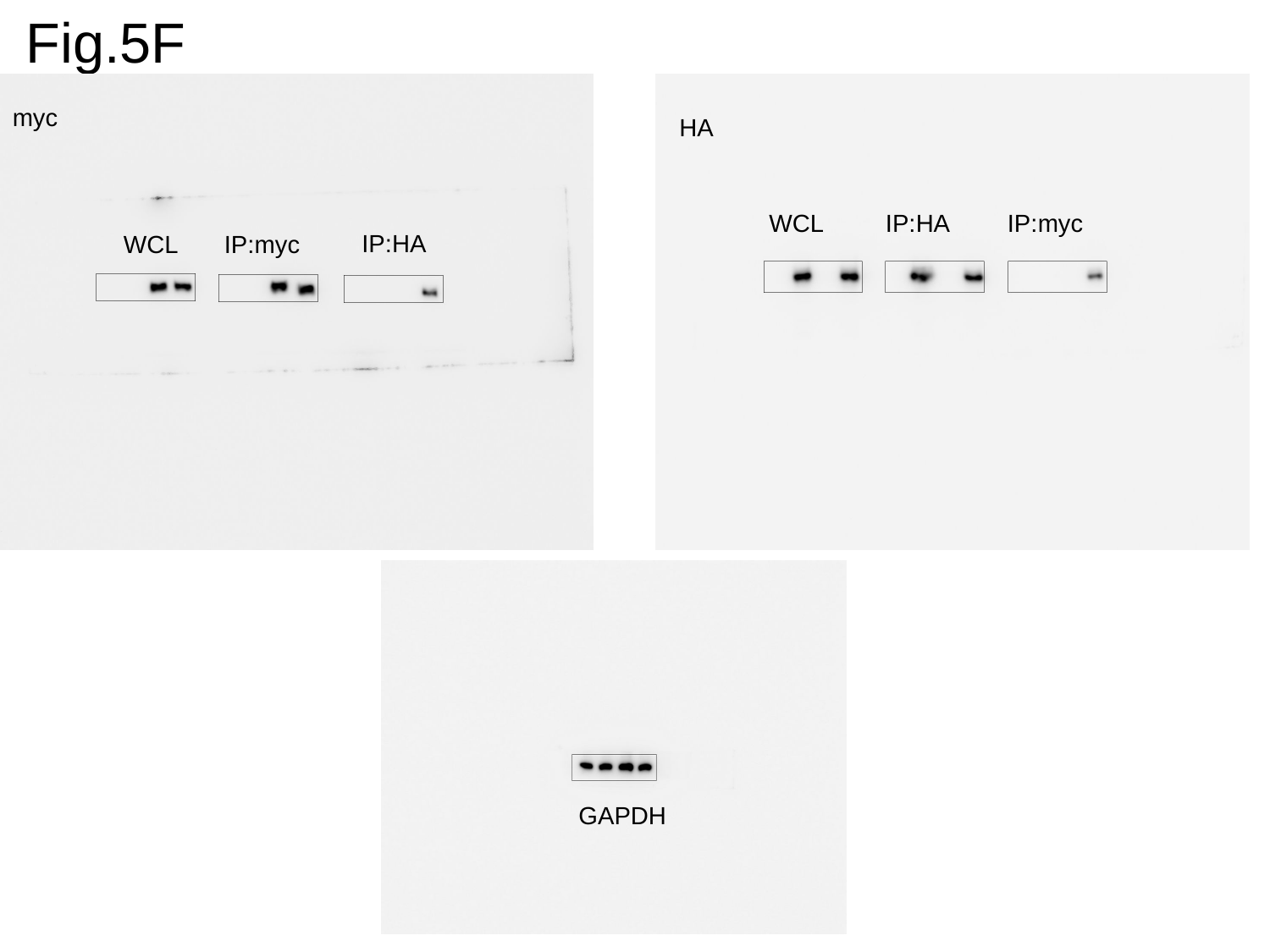

Fig.5F
myc
HA
IP:myc
WCL
IP:HA
IP:HA
WCL
IP:myc
GAPDH

## Slide 6
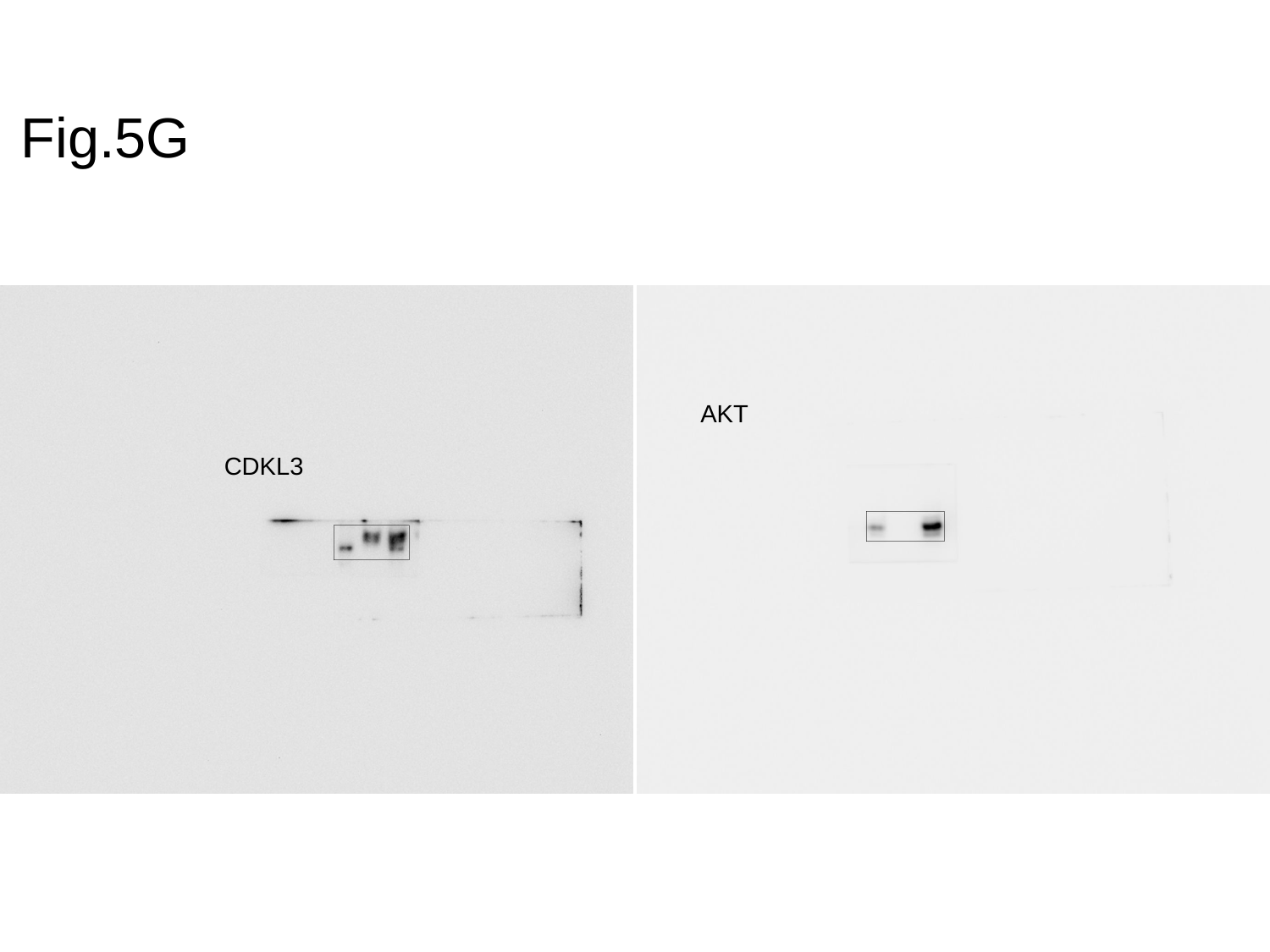

Fig.5G
AKT
CDKL3

## Slide 7
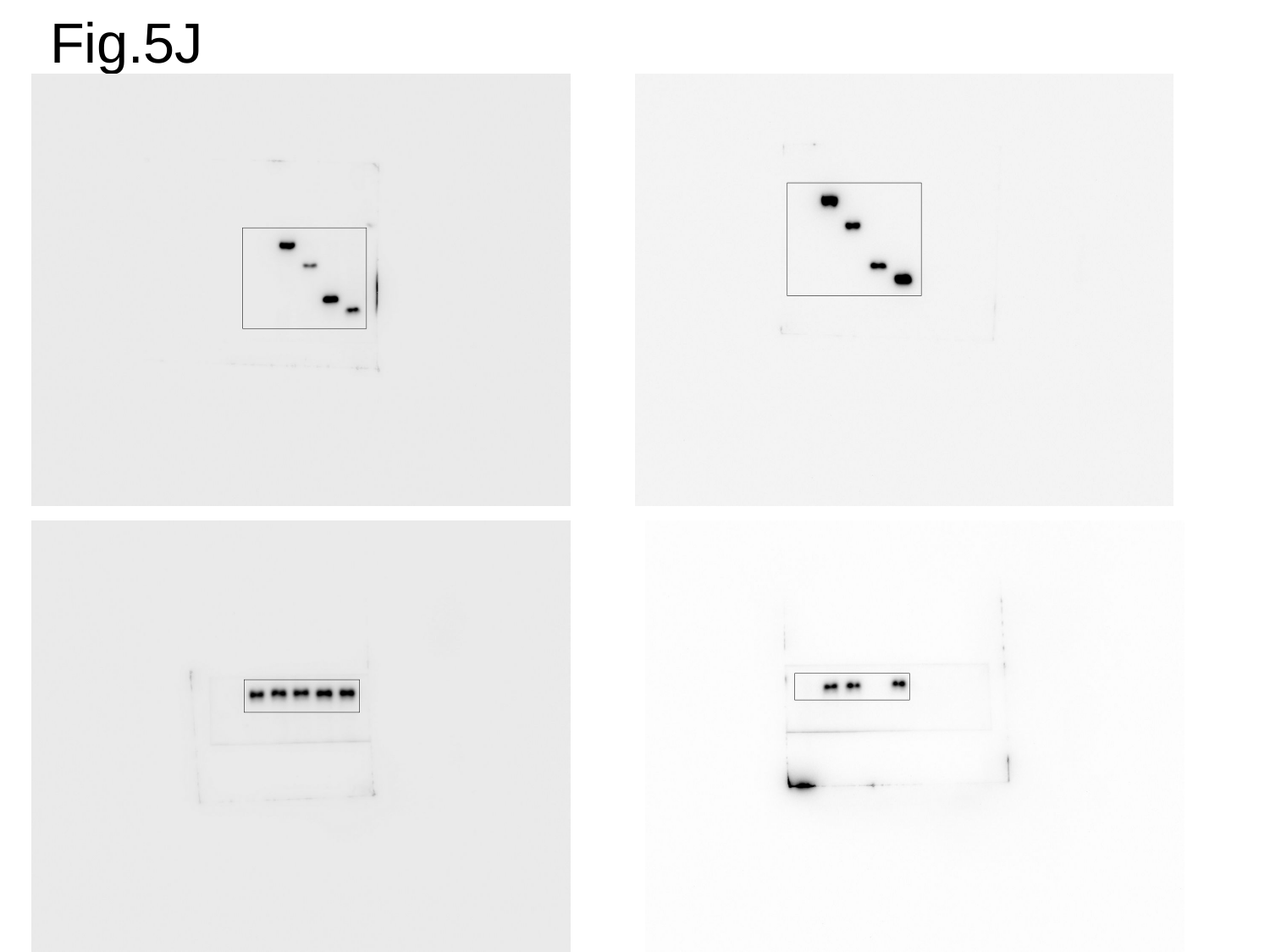

Fig.5J

## Slide 8
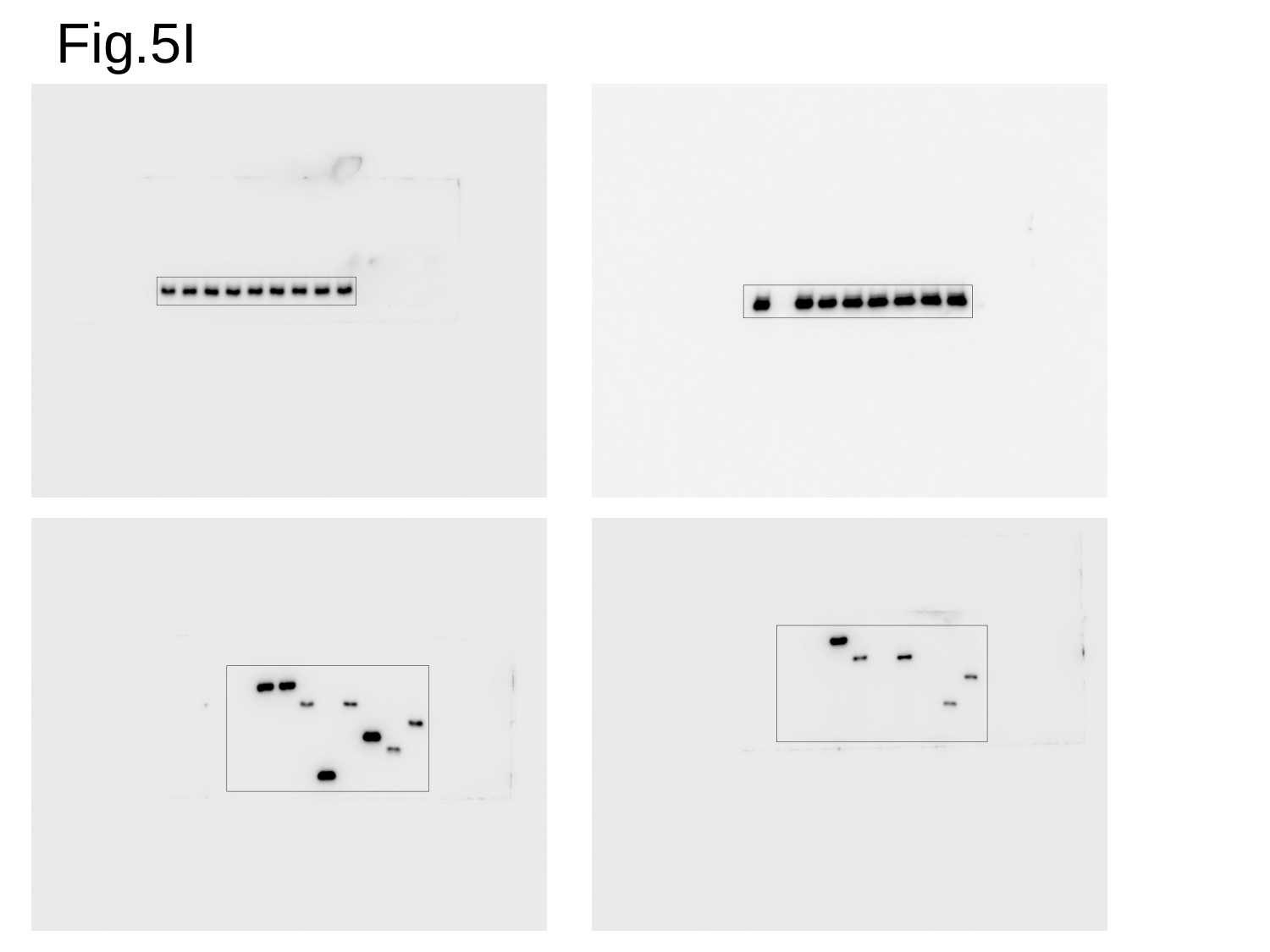

Fig.5I

## Slide 9
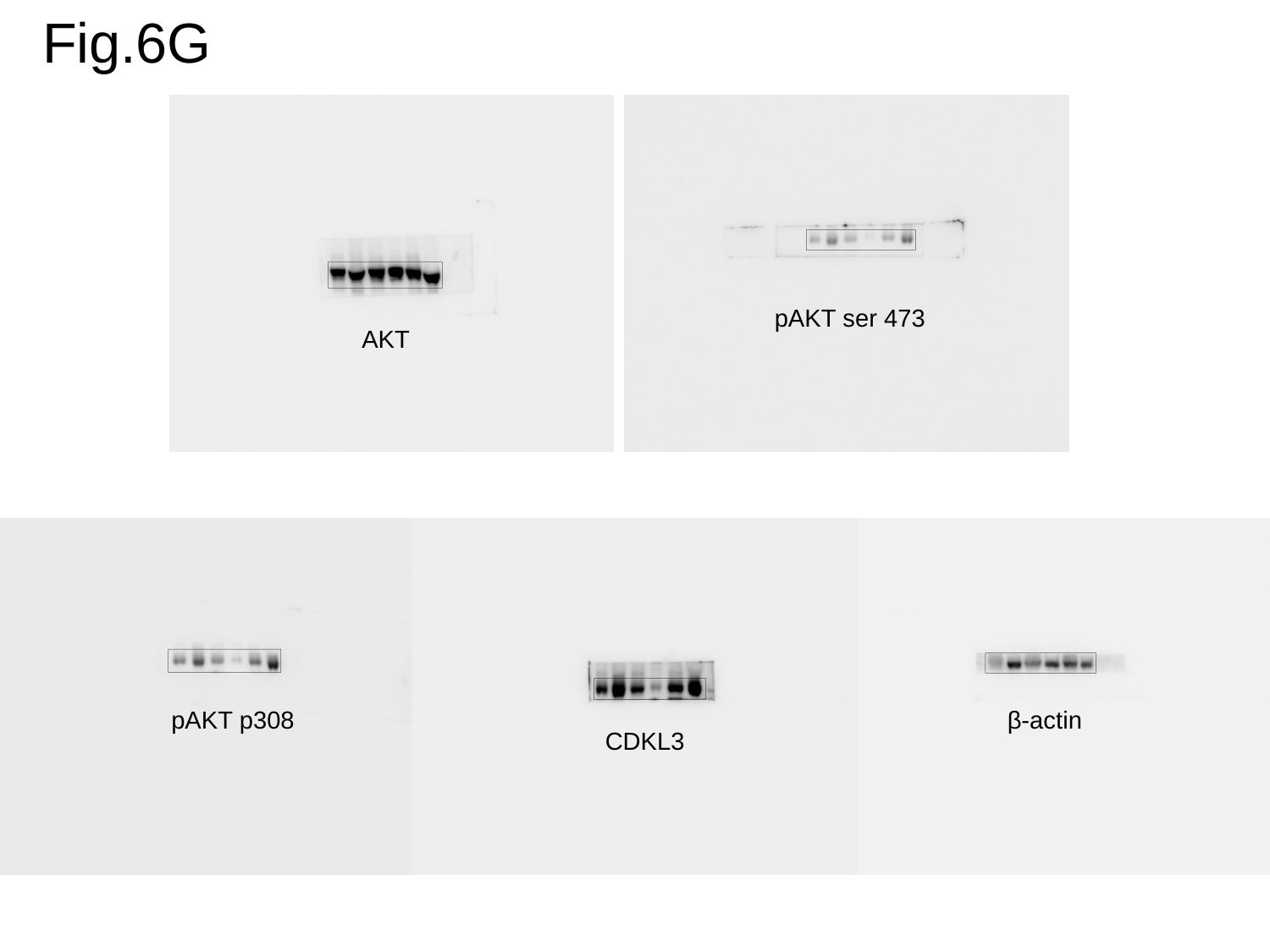

Fig.6G
pAKT ser 473
AKT
pAKT p308
β-actin
CDKL3

## Slide 10
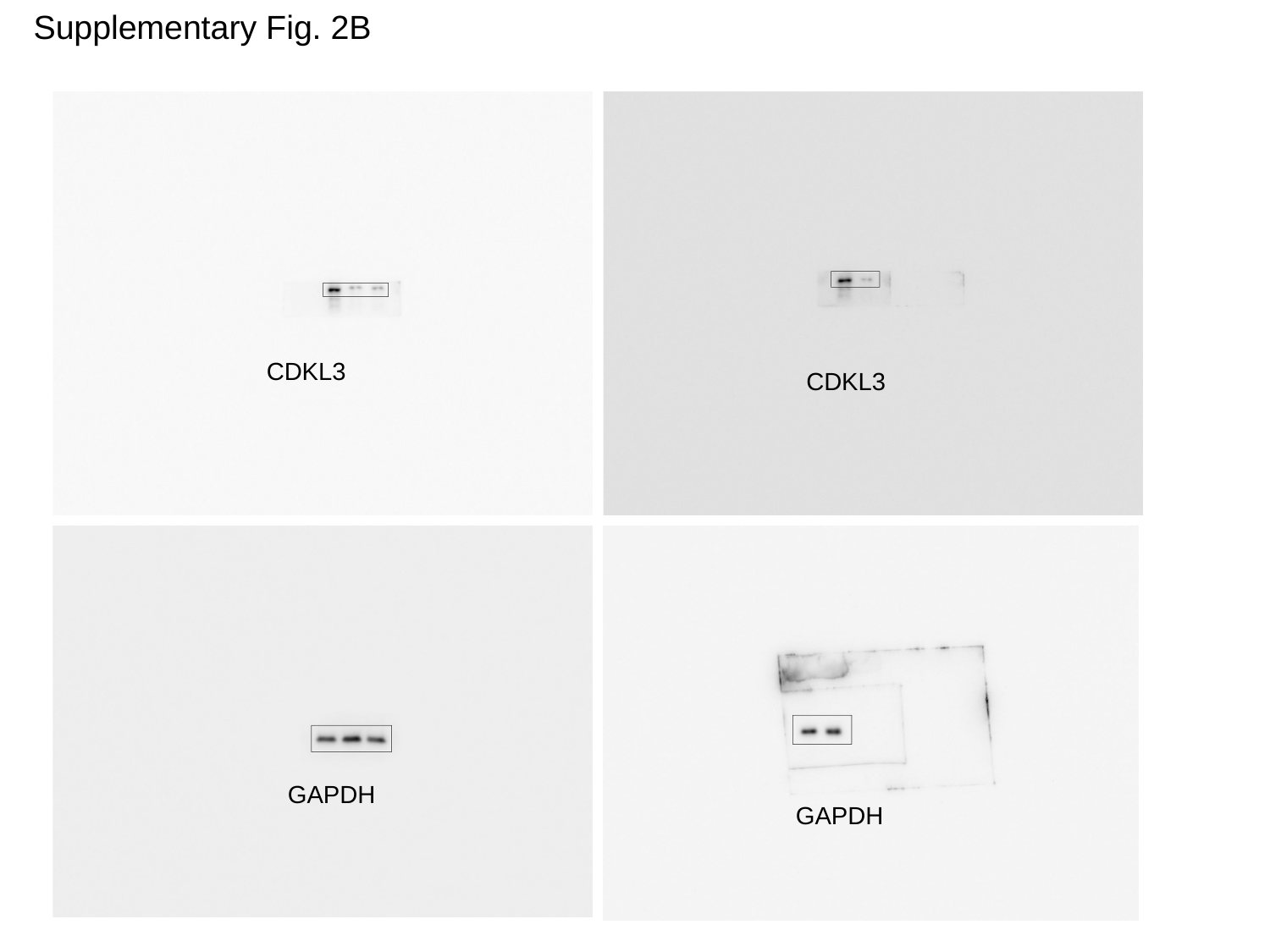

Supplementary Fig. 2B
CDKL3
GAPDH
CDKL3
GAPDH

## Slide 11
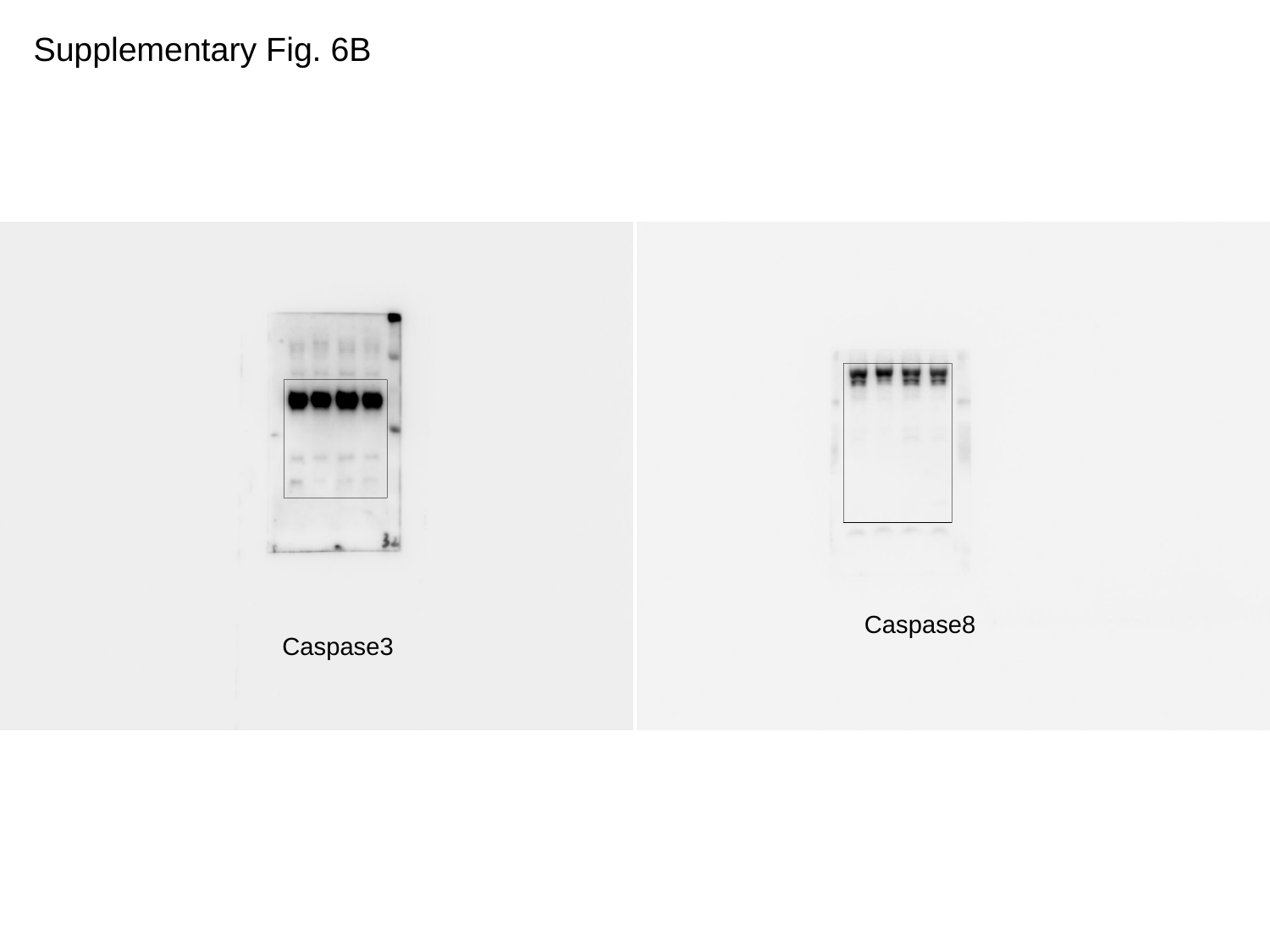

Supplementary Fig. 6B
Caspase8
Caspase3

## Slide 12
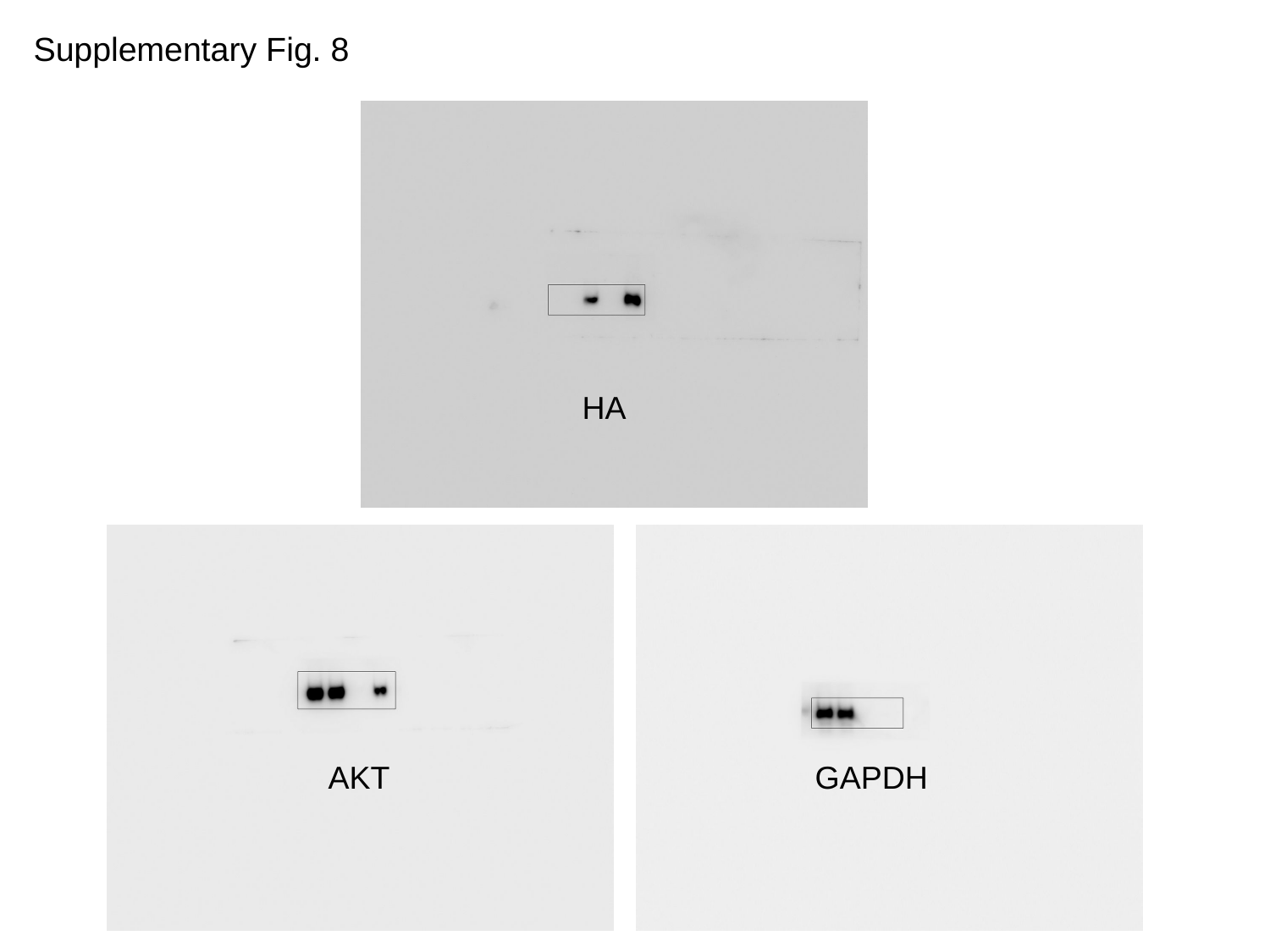

Supplementary Fig. 8
 HA
 AKT
 GAPDH

## Slide 13
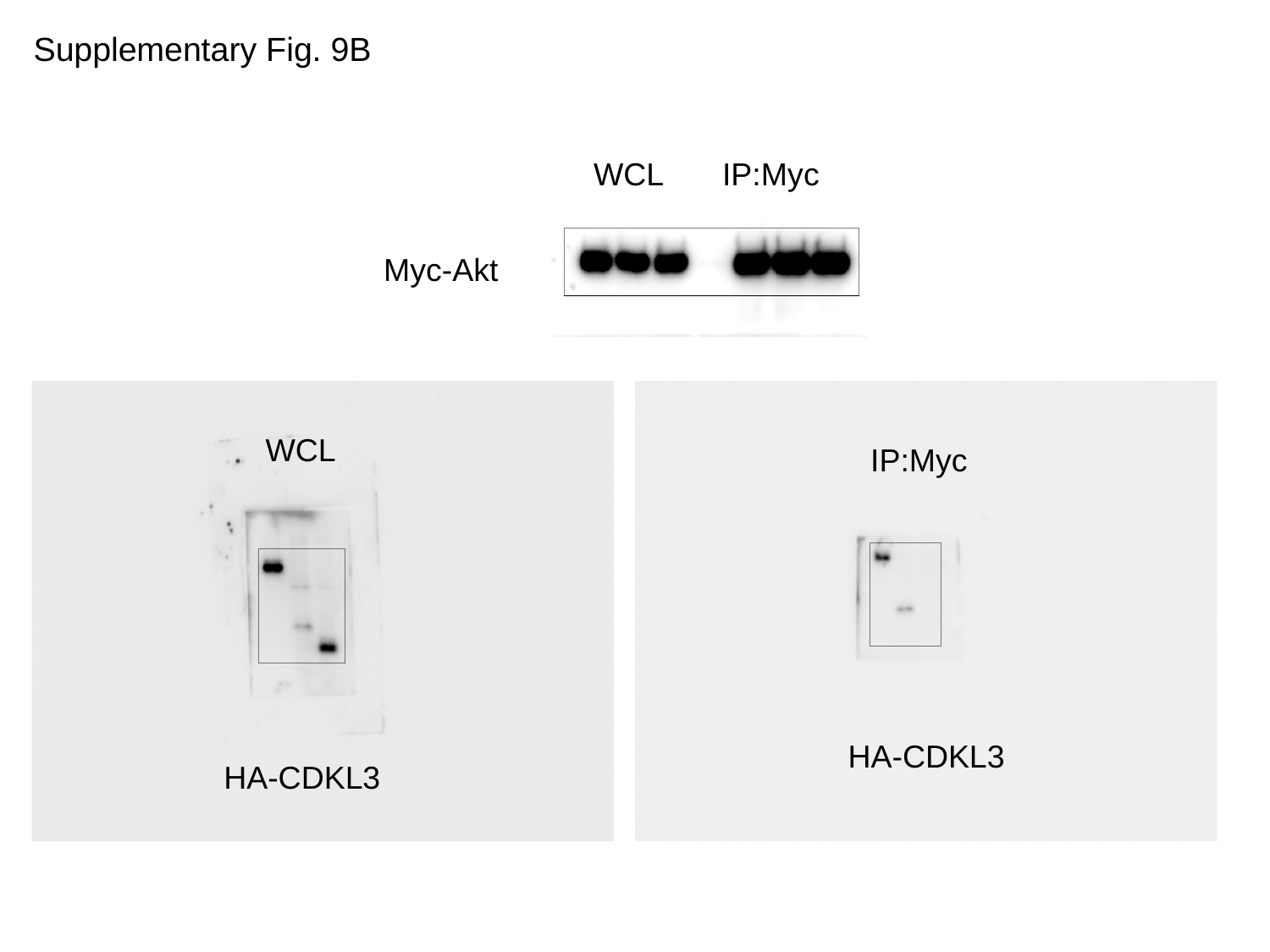

Supplementary Fig. 9B
 WCL
IP:Myc
Myc-Akt
 WCL
IP:Myc
 HA-CDKL3
 HA-CDKL3
