# Supplementary material for: CDKL3 promotes osteosarcoma progression by activating Akt/PKB
Source: Life Sci Alliance. 2020 Mar 31;3(5):e202000648. doi: 10.26508/lsa.202000648 (PMC7119369; doi:10.26508/lsa.202000648)
Supplement: Supplementary file 3 [file LSA-2020-00648_TableS2.docx]

**Supplementary Table 3.** CDKL3^-/-^ cell lines information

|  | Nucleic Acid | InDel | Amino Acid |
| --- | --- | --- | --- |
| **CDKL3 gRNA** | GGGCTGTATGATCATTGAGA | | |
| **Wild type** | CCTGTGGATATCTGGGCTTTGGGCTGTATGATCATTGAGATGGCCACTGGAAATCCCTAT |  | PVDIWALGCMIIEMATGNPY |
| **CDKL3^-/-^ U2OS** | CCTGTGGATATCTGGGCTTTGGGCTGTATGATCATTXXXXXXXXXXXTGGAAATCCCTAT | -11 | PVDIWALGCMIIWKSL |
|  | CCTGTGGATATCTGGGCTTTGGGCTGTATGATCATXXXXXTGGCCACTGGAAGTCCCTAT | -5 | PVDIWALGCMIIGHWKSL |
| **CDKL3^-/-^ Saos-2** | CCTGTGGATATCTGGGCTTTGGGCTGTATGATCATTTCCAGATGGCCACTGGAAATCCCTAT | -1+3 | PVDIWALGCMIISRWPLEIP |
|  | CCTGTGGATATCTGGGCAGGCTGTGGATATCCAGATGGCCACTGGAAATCCCTAT | -20+15 | PVDIWAGCGYPDGHWKSL |
