# Supplementary material for: Correction: CDKL3 promotes osteosarcoma progression by activating Akt/PKB
Source: Life Sci Alliance. 2022 Oct 21;5(11):e202201656. doi: 10.26508/lsa.202201656 (PMC9603691; doi:10.26508/lsa.202201656)
Supplement: Reviewer comments [file LSA-2022-01656_review_history.pdf]

Life Science Alliance

Correction: CDKL3 promotes osteosarcoma progression by activating Akt/PKB

Aina He, Lanjing Ma, Yujing Huang, Haijiao Zhang, Wei Duan, Zexu Li, Teng Fei, Junqing Yuan, Hao Wu, Liguo Liu, Yueqing Bai, Wentao Dai, Yonggang Wang, Hongtao Li, Yong Sun, Yaling Wang, Chunyan Wang, Ting Yuan, Qingcheng Yang, Songhai Tian, Min Dong, Ren Sheng, and Dongxi Xiang
DOI: https://doi.org/10.26508/lsa.202201656

Corresponding author(s): Dongxi Xiang, Shanghai Research Center of Biliary Tract D

Review Timeline:

Submission Date:	2022-09-14
Accepted:	2022-09-21

Scientific Editor: Eric Sawey, PhD

Transaction Report:

(Note: With the exception of the correction of typographical or spelling errors that could be a source of ambiguity, letters and reports are not edited. The original formatting of letters and referee reports may not be reflected in this compilation.)

September 21, 2022

RE: Life Science Alliance Manuscript #LSA-2022-01656

Dr. Dongxi Xiang
Shanghai Research Center of Biliary Tract D
Medicine
77 Avenue Louise Pasteur
NRB458
Boston, MA 02115

Dear Dr. Xiang,

Thank you for submitting your Correction entitled "Correction: CDKL3 promotes osteosarcoma progression by activating Akt/PKB".

IMPORTANT: If you will be unreachable at any time, please provide us with the email address of an alternate author. Failure to respond to routine queries may lead to unavoidable delays in publication.

You can contact the journal office with any questions, contact@life-science-alliance.org

Sincerely,

Eric Sawey, PhD
Executive Editor
Life Science Alliance
http://www.lsajournal.org

September 21, 2022

RE: Life Science Alliance Manuscript #LSA-2022-01656

Dr. Dongxi Xiang
Shanghai Research Center of Biliary Tract D
Medicine
77 Avenue Louise Pasteur
NRB458
Boston, MA 02115

Dear Dr. Xiang,

Thank you for submitting your Correction entitled "Correction: CDKL3 promotes osteosarcoma progression by activating Akt/PKB".

IMPORTANT: If you will be unreachable at any time, please provide us with the email address of an alternate author. Failure to respond to routine queries may lead to unavoidable delays in publication.

You can contact the journal office with any questions, contact@life-science-alliance.org

Sincerely,

Eric Sawey, PhD
Executive Editor
Life Science Alliance
http://www.lsajournal.org
